# Effect of Manganese on the Strength–Toughness Relationship of Low-Carbon Copper and Nickel-Containing Hull Steel

**DOI:** 10.3390/ma17051012

**Published:** 2024-02-22

**Authors:** Zhide Zhan, Zhongran Shi, Zemin Wang, Wenjing Lu, Zuoning Chen, Dian Zhang, Feng Chai, Xiaobing Luo

**Affiliations:** 1 School of Materials Science and Engineering, Shanghai Institute of Technology, 100 Haiquan Road, Shanghai 201418, China; 15300595562@163.com (Z.Z.); luwenjing11741@163.com (W.L.); 2 Institute of Structural Steels, Central Iron and Steel Research Institute, Beijing 100081, China; 18764374757@163.com (Z.C.); zd995270508@163.com (D.Z.); chaifeng@cisri.com.cn (F.C.); luoxiaobing@cisri.com.cn (X.L.); 3 The State Key Laboratory of Refractories and M1, The State Key Laboratory of Refractories and Metallurgy, Wuhan University of Science and Technology, China Metallurgy, Wuhan 430081, China

**Keywords:** reversed austenite, tempering, deformation twin, mechanical properties

## Abstract

The influence of varying the manganese (Mn) contents of high-strength copper-containing hull steel on its microstructural evolution and mechanical properties was investigated. With increasing Mn content from 2 to 5%, the tensile strength of the steel increased by ~100 MPa, while the elongation of steel remained at ~23.5%, indicating good plasticity. However, the 2Mn sample had 128 J higher low-temperature (−84 °C) impact work than the 5Mn sample. The microstructures of different Mn steels were composed of fresh martensite (FM), ferrite/tempered martensite (F/TM), and reversed austenite (RA). The increase in Mn content markedly increased the presence of RA and intensified the work hardening caused by the transformation-induced plasticity (TRIP) effect during the tensile process. However, as the phase transformation in different Mn steels occurred in the early stage of strain and did not extend throughout the entire plastic deformation process, increasing plasticity via phase transformation was difficult. In addition, although the volume fraction of RA increased significantly in 4Mn and 5Mn steels, the stability of RA significantly decreased. The presence of numerous metastable blocks and coarse lath-like RA contributed little to low-temperature impact work and was even detrimental to toughness. The substantial fresh martensite resulting from phase transformation facilitated microcrack generation, owing to rapid volume expansion and mutual impacts, thus reducing the work required for crack formation. Additionally, the abundance of deformation twins significantly reduced the work needed for crack propagation. These combined actions significantly reduced the low-temperature toughness of 4Mn and 5Mn steels.

## 1. Introduction

With the development of marine resources, advanced marine equipment has become indispensable. Using hull steel with high strength, high toughness, and high weldability has become a trend in marine engineering [1]. However, achieving these properties often involves the addition of costly alloying elements, such as Ni and Mo, to the traditional alloy composition. This practice, while effective, is expensive and limits broader applications. Medium-manganese (Mn) steel, the third generation of advanced high-strength steel, has attracted wide attention. The underlying design concept of replacing nickel with low-cost Mn has broad application prospects. Through high-temperature tempering, a substantial quantity of metastable reversed austenite (RA) can be introduced into the microstructure, enabling its retention at room temperature. The size, distribution, stability, and content of this austenite affects the mechanical properties of the steel [2,3,4,5]. The introduction of RA necessitates high-temperature tempering, which will soften the ferrite matrix and reduce the strength of the material. Consequently, a current focal point in research is enhancing material strength while preserving plasticity. Numerous studies have indicated that substituting ferrite (F) with fresh martensite (FM) significantly improves tensile strength. Some researchers have employed novel critical interval annealing and multistep partition heat treatments to replace the soft phase of ferrite with FM, resulting in a 300 MPa increase in tensile strength [6,7]. Wang [8] increased the volume fraction of FM from 9.8 to 57.8%, increasing its contribution to tensile strength from 13.7 to 68.8%. This adjustment led to the highest recorded tensile strength of 1150 MPa.

The introduction of FM in medium- and low-Mn steel primarily occurs through the phase transformation of RA. Mn had a stronger effect on stabilizing austenite than Ni [9]. The presence of Mn significantly influences the formation of metastable austenite. Experimental findings from the relevant literature [10,11,12] suggest that elements such as carbon (C), Mn, and other austenite-stabilizing elements tend to distribute into the RA phase. Within a short isothermal annealing time (a few minutes), Mn can be distributed between ferrite and austenite. Sun et al. [13] analyzed the microstructure characteristics of cold-rolled experimental steel with 0.2% carbon, 7/10% Mn, and 3% aluminum and found that the retained austenite content increased from 30 to 50% with the increase in the Mn content from 7 to 10%. However, this increase led to reduced austenite stability. Shi [14] investigated the tensile properties and strain-hardening behavior of Fe–(0.1–0.4) C–(5–7) Mn steel. The tensile strength exhibited a 470 MPa increase when the Mn content increased from 5 to 7%, which was accompanied by an elevation in the RA content from 34 to 45%. Morawiec [15] compared two medium manganese sheet steels (3Mn–Al and 5Mn–Al) that were alloyed. The results showed that the mechanical properties of 3Mn steel were more strain rate sensitive than those of 5Mn steel. The 3Mn–Al steel is characterized by better total elongation, due to a larger fraction of retained austenite and a more pronounced transformation-induced plasticity effect. Multi-phase microstructures were produced in these steels, and the multi-phase microstructures had higher yield strength compared to single-phase (austenite) microstructures [16].

Currently, much of the research in Mn steel focuses on enhancing its strength and plasticity [17,18,19,20,21,22]. However, studies examining low-temperature toughness, particularly the impact of RA phase transformation on toughness, are relatively scarce. It is widely believed that slender, thin-film, or lath-like RA can enhance low-temperature toughness by deflecting cracks owing to its high stability [23,24]. Metastable RA, despite its poor stability, absorbs energy through phase transformation and alleviates stress concentration [25]. Huang [26] subjected 0.12C–3.0Mn steel to a three-step heat treatment method of complete quenching + two-phase quenching + critical zone quenching to achieve an impact work of 200 J at −80 °C. Liu [27] achieved good low-temperature impact toughness (>90 J at 77 K) by designing metastable austenite with a core–shell structure and specific volume fraction in maraging steel. Kwok [28] were designed two medium Mn steels with 0.2 and 0.5 wt pct C. The fracture surfaces were investigated and the Transformation Induced Plasticity (TRIP) effect was found to occur more readily in the Low C Charpy specimen. The low C steel had a corrected Charpy impact energy (KV10) of 320 J cm (−2) compared to 66 J cm (−2) in the high C steel, despite both having a ductility of over 35 pct. Mohapatra [29] investigated the influence of annealed temperature on microstructural and mechanical property Fe-6.22Mn-0.18C steel. An excellent strength–ductility combination was found at 650 °C.

According to the above discussion, most studies on Mn steels focused on those with Mn contents of 5% and higher and mainly investigated the tensile properties of the steel. Only a few studies have investigated the effect of Mn content on the low-temperature toughness and microstructural evolution of medium- and low-Mn steel, with an Mn content of less than 5%. Particularly, the relationship between Mn content and the change in the microstructure properties of medium- and low-Mn steel remains ambiguous. In this study, four kinds of Mn steel were produced. The purpose of this paper is to reveal the correlation between Mn content and microstructural changes and explain the influence of Mn on the strength–toughness relationship. In addition, the phenomenon of deformation twins in the test steel during low-temperature impact is explored, which has not received enough attention in the previous research on Mn steel. This work provides an important reference for further alloy design of medium- and low-Mn steel.

## 2. Experimental Procedure

**Figure 1:**
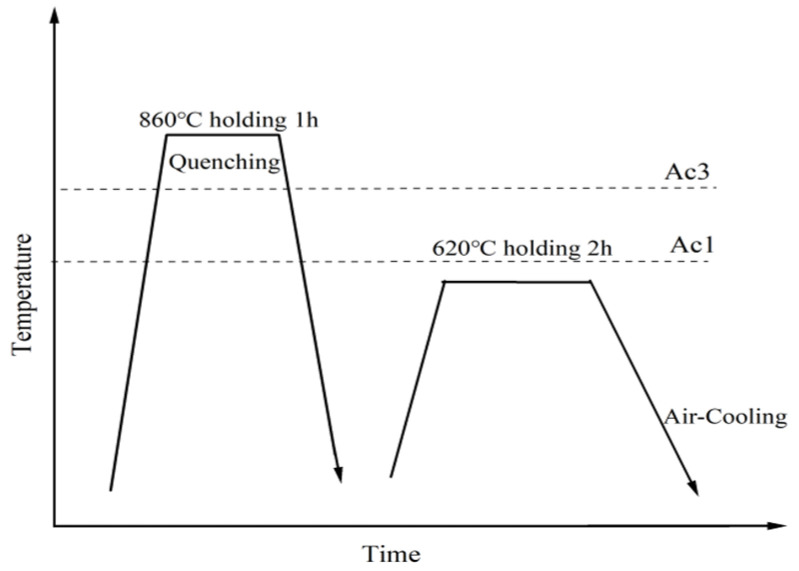
Schematic diagram of the heat treatment process.

Experimental steels with different manganese contents were chosen for this study, of which the chemical compositions are listed in Table 1. The four steels were designated as ‘2Mn’, ‘3Mn’, ‘4Mn’ and ‘5Mn’steels, respectively. The experimental steels were melted using a vacuum induction furnace, and then cast as 50 kg ingots, before finally being forged to the slab billet (150 × 110 × 60 × mm^3^). Thereafter, the steels had experienced similar thermomechanical processing conditions in the two-stage controlled rolling process; that is, the slab billets were reheated to 1200 °C, held for 2 h in a resistance-heated furnace, the start rolling temperature of rough rolling and of the second stage were 1150 °C and 950 °C, respectively, and the finish rolling temperature was 870 °C. After hot rolling, the steel plates (12 mm) were immediately air-cooled to an ambient temperature. A thermal expansion sample (φ 3 mm × 10 mm) was extracted from the steel plate. The start temperature (Ac1) and end temperature (Ac3) of austenite transformation and the start temperature (Ms) and end temperature (Mf) of martensite transformation were measured using the Formaster-FII automatic phase change instrument (Table 2). According to the measured phase-transition points, heat treatment parameters were established through quenching (860 °C) followed by tempering (620 °C), as illustrated in Figure 1.

The Charpy V-notch standard sample of 10 mm × 10 mm × 55 mm was used as the impact specimen. The low-temperature (−40, −60, and −84 °C) toughness test was conducted using a JBN-300C (Shanghai Huayan Instrument Equipment Co., Ltd., Shanghai, China) pendulum impact testing machine. The −60 °C sample was subjected to an oscilloscope impact test, and the force–displacement–work curve was recorded. For the tensile tests, samples were extracted perpendicular to the rolling direction, and the sample standard was 5 mm in diameter and 25 mm in gauge. The room-temperature tensile test was performed using the GNT microcomputer-controlled electronic universal testing machine (Ncs Testing Technology Co., Ltd., Beijing, China). The tensile rate was set at 0.00025/s until the yield point was reached, and was subsequently increased to 6 mm/min. For microstructural analysis and observation of impact fractures, a small portion of the heat-treated plate underwent mechanical grinding and polishing. This prepared specimen was then etched using a 4% nitric acid alcohol solution for 15 s. Observations were conducted using a Quanta650 field (Thermo Fisher Scientific, Waltham, MA, USA)-emission scanning electron microscope. Electron backscatter diffraction (EBSD) analysis was performed using the EDAX Velocity Super ultra (EDAX Inc, Santa Clara, CA, USA)-fast EBSD probe attached to JSM 7200F (the electrolytic polishing solution was 6% perchloric acid, the voltage was 25 V, and the electropolishing duration was 10 s). The residual austenite of the sample was detected using a D8 ADVANCE (Bruker Corporation, Bilerica, MA, USA) X-ray diffractometer (XRD) with a Co target, tube current of 40 mA, tube voltage of 35 kV, scanning speed of 2°/min, and Lynxeye XE detector. The volume fraction of residual austenite (*Vγ*) in the sample was calculated using Formula (1) [30]:

*Vγ* = 1.4 *Iγ*/(1.4 *Iγ* + *Iα*) (1)

where *Iγ* is the integral intensity of (200) *γ*, (002) *γ*, (220) *γ*, and (311) *γ* diffraction peaks of austenite, and *Iα* denotes the integral intensity of the (101) *α*, (200) *α*, and (211) *α* diffraction peaks of ferrite. Samples for the XRD test were prepared via the same method adopted for EBSD observation. The sample scanning area of 10 cm × 10 cm was used as the XRD test area. The distribution of reversed austenite and the enrichment of elements were observed and characterized by a JEM-F200 (Japan Electronics Co., Ltd., Tokyo, Japan) cold field emission transmission electron microscope (TEM), with an accelerating voltage of 200 kV and self-contained 100 mm^2^ electric refrigeration energy-dispersive spectrometer (EDS).

## 3. Results

### 3.1. Mechanical Properties

**Figure 2:**
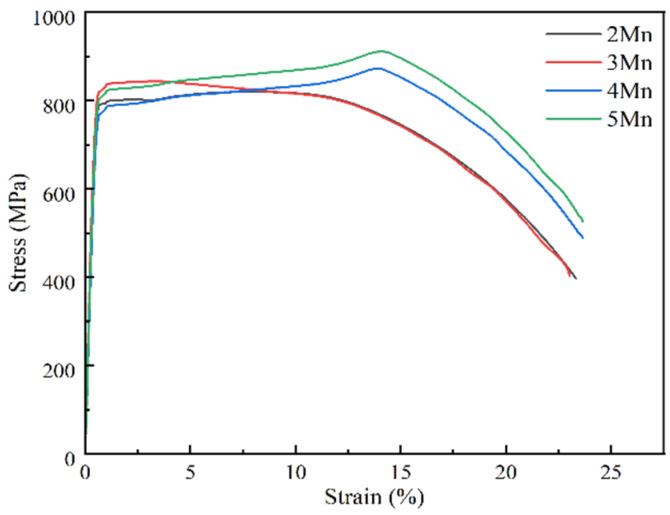
Engineering stress–strain curves of experimental steels.

Figure 2 depicts the tensile engineering stress–strain curve of the test steel. Various test steels exhibit discontinuous yielding [31]. In dual-phase steel, this phenomenon is primarily due to dislocation pinning after the precipitation of aging–strengthening alloying elements such as Cu. This process leads to the formation of carbon and nitrogen atom clusters, known as the “Cottrell atmosphere,” caused by the “pinning and anchoring” effect. Table 3 shows the yield strength (Rp0.2), tensile strength (Rm), elongation after fracture (A), reduction in area (Z), and other parameters of the specimen. The tensile strength and yield strength of the samples increase continuously with the increase in Mn content, and the increase in tensile strength is more pronounced. The increase in the Mn content also leads to a slight decrease in the yield ratio, but at 5Mn, the yield ratio is maintained at ~0.9. With the increase in Mn content, the elongation of the tested steels with different Mn contents is basically maintained at ~23.5%, while the Z decreases slightly from 79% to 75%. Overall, these steels exhibit good plasticity. In addition, the dual-phase steel yield strength depends on the strength of the soft phase, which in our research is RA. The change in yield strength is not significant. With the increasing of Mn content, although the increase in solid solution Mn content enhances the effect of solid solution strengthening, the grain size of RA increases, reducing the effect of fine grain strengthening. Under the combined influence of these two factors, the change in yield strength is less significant.

Table 4 presents the RA content and low-temperature impact work of the test steel. The rise in Mn content corresponds to a gradual increase in RA content, which reaches 43% at 5Mn. This suggests that even at higher content levels, the RA maintains relative stability, showing minimal transformation into FM or ε-martensite during tempering cooling. However, the low-temperature impact work gradually decreases as the Mn content increases. At various low temperatures, the impact work decreases by over 120 J with the increase from 2Mn to 5Mn, exhibiting an opposite trend to that of RA. Similar observations have been reported in the relevant literature [32,33,34]. This anomaly is primarily associated with the morphology and stability of RA. Furthermore, the decrease in impact work in this test steel is attributed to Mn segregation at grain boundaries, a factor to be further explored in the subsequent analysis.

**Figure 3:**
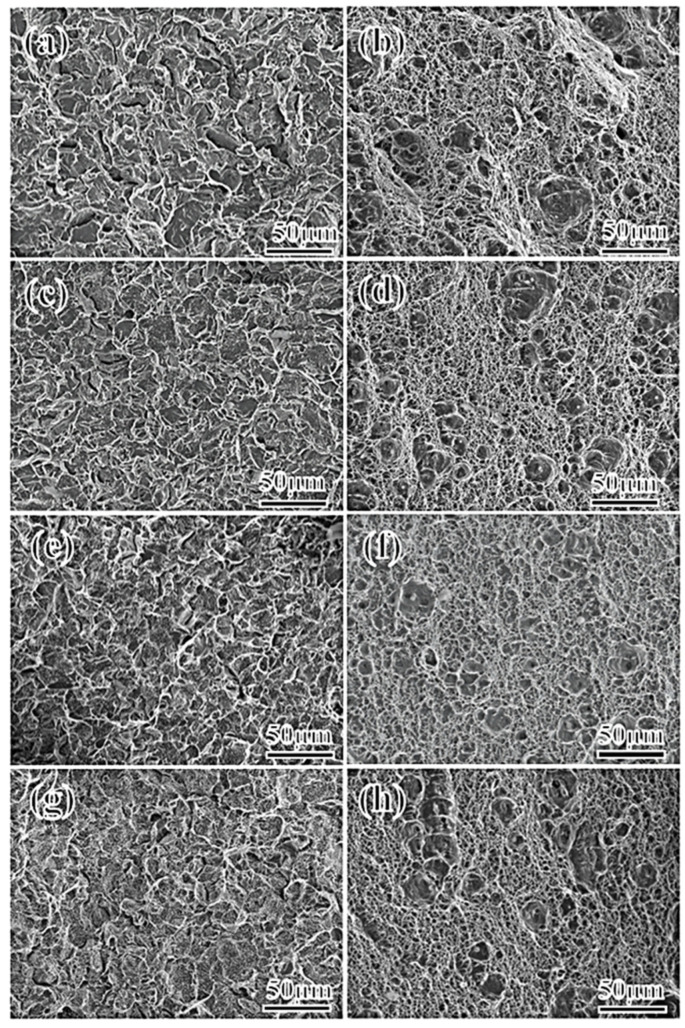
Fracture morphology of impact specimens of steel with different Mn content at 2Mn (**a**,**b**), 3Mn (**c**,**d**), 4Mn (**e**,**f**), and 5Mn (**g**,**h**).

Figure 3 illustrates the fracture morphology of the steel fiber zone and the quasi-cleavage zone at −60 °C. The diagram reveals that at a low Mn content (2–3%), the fiber area exhibits numerous large and deep dimples. The fracture predominantly demonstrates ductile characteristics, occupying 71 and 62% of the area, with the remaining area displaying quasi-cleavage fracture. The quasi-cleavage zone exhibits small cleavage planes, additional tearing ridges, and tongue-like patterns. Conversely, at higher Mn contents (4–5%), the size of dimples in the fiber area decreases, resembling the morphology of the low-Mn sample. However, the area ratio significantly reduces to approximately 35 and 22%. The dominant fracture mode becomes quasi-cleavage, with a noticeable increase in tongue-like patterns. The quasi-cleavage fracture mode is dominant. These tongue-shaped features, observed as bosses or pits in the crystal, result from the challenges posed by critical deformation under impact loads. Crystal deformation occurs through deformation twinning to form twins, and the fracture characteristics exhibit pronounced tongue-like patterns.

### 3.2. Oscillographic Shock

**Figure 4:**
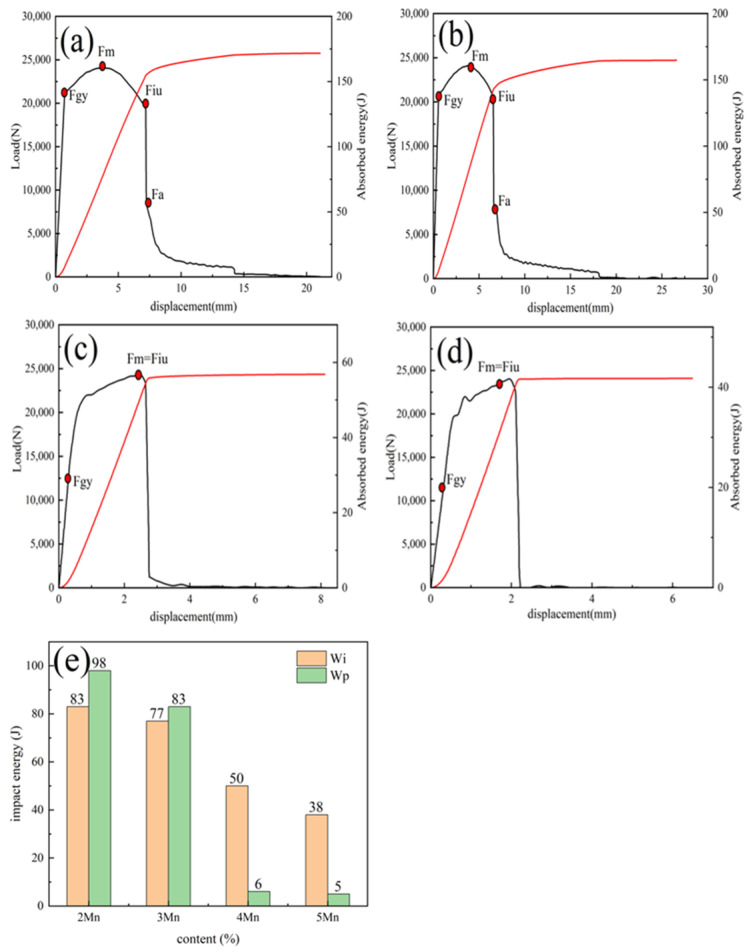
Load and absorbed energy vs. displacement curves with samples of 2Mn (**a**), 3Mn (**b**), 4Mn (**c**), and 5Mn (**d**); (**e**) part characteristic value of the oscillographic impact curve of steel with different Mn contents. Note: Fgy—yield force, Fiu—Unstable crack propagation initiation force, Fa—Unstable crack propagation termination force, Wi—crack initiation energy, Wp—crack propagation energy, Fm—maximal impact force, Sm—maximum load displacement.

**Figure 5:**
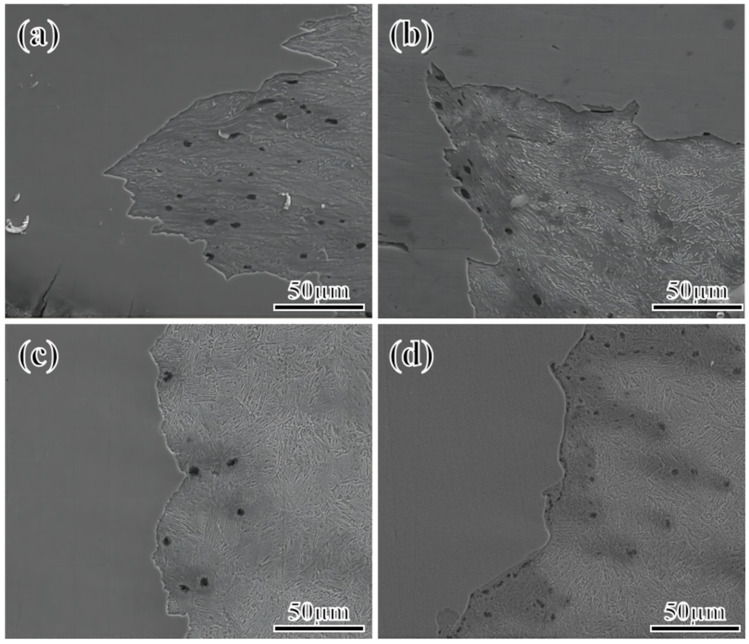
The crack propagation path of steel with different Mn content of samples at 2Mn (**a**), 3Mn (**b**), 4Mn (**c**), and 5Mn (**d**).

Figure 4 further clarifies the difference between the crack formation stage and the crack propagation stage of the test steel under varying Mn content and illustrates the difficulty of crack initiation during the propagation process. The displacement–load and displacement–absorption energy curves at −60 °C for the test steel with varying Mn content were recorded and analyzed. Several eigenvalues were also recorded, as depicted in Figure 4e. At lower Mn content (2–3%), more energy is required for crack initiation and propagation, making crack formation more difficult and establishing a stronger hindrance effect in the subsequent propagation stage. The crack exhibits a certain steady-state propagation stage characterized by higher steady-state propagation energy (approximately 80–90 J), followed by subsequent instability, indicative of a higher toughness level. At higher Mn content (4–5%), significantly less energy is required for crack initiation and propagation; particularly, the crack propagation energy decreases significantly. This suggests that unstable propagation occurs soon after crack formation, and only a small amount of crack-tearing energy is generated. Considering the significant variance in crack propagation energy among steels with different Mn contents, a detailed investigation into the variation in crack propagation paths among these test steels was conducted. Figure 5 shows the scanning electron microscopy image of the side view of the impact fracture surface of 2–5Mn test steel. With the increase in the Mn content, the crack propagation path gradually changes from bent to straight, which indicates a gradual weakening in the effect of certain factors hindering crack propagation.

### 3.3. Microstructural Characterization

**Figure 6:**
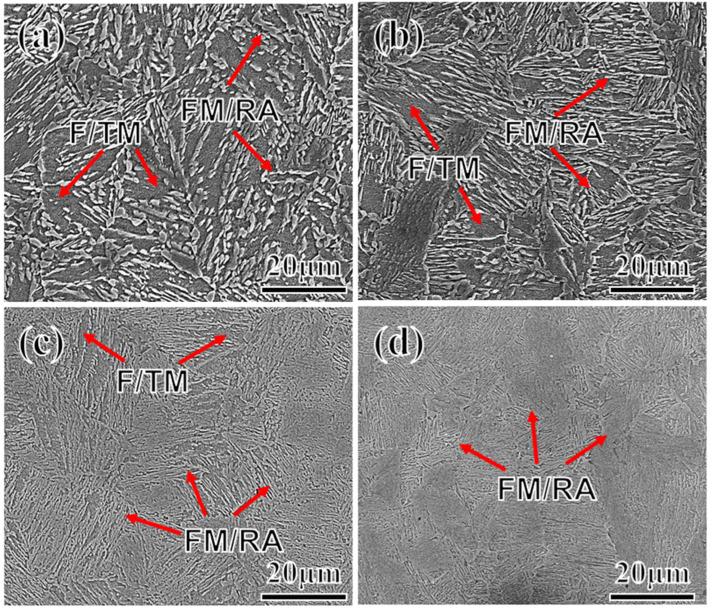
SEM images with samples of 2Mn (**a**); 3Mn (**b**); 4Mn (**c**); and 5Mn (**d**). (signaled by the red arrows:‎ FM: fresh martensite, F: ferrite, RA: reverse austenite, TM: tempered martensite).

Figure 6 displays the scanning electron microscopy image of the test steel. The microstructures of the tempered test steels with different Mn contents are composed of FM, RA, and F/TM. The brighter convex regions in the figure depict the mixed area of RA and FM. FM begins to form on the habit plane of RA through a non-diffusive phase transformation, maintaining shear coherence; therefore, distinguishing between FM and RA under a scanning electron is difficult and may require more refined microscopic observation. Moreover, with the increase in the Mn content, the white convex area, FM/RA, increases significantly. Particularly at 5Mn (Figure 6d), this area almost encompasses the entire region. This expansion occurs mainly because Mn reduces the phase-transition point of the test steel. At the same tempering temperature, samples with higher Mn content enter the two-phase region earlier. Owing to structural and energy fluctuations, some sections of the sample might enter the two-phase region and undergo phase transition, even if the tempering temperature is below Ac1. The darker regions indicate tempered martensite, some of which transform into ferrite (F) due to carbon diffusion. The darkness in these areas arises from carbon and alloying element enrichment during the transition from martensite to austenite. This enrichment results in lower alloy element content, making these areas more vulnerable to corrosion.

**Figure 7:**
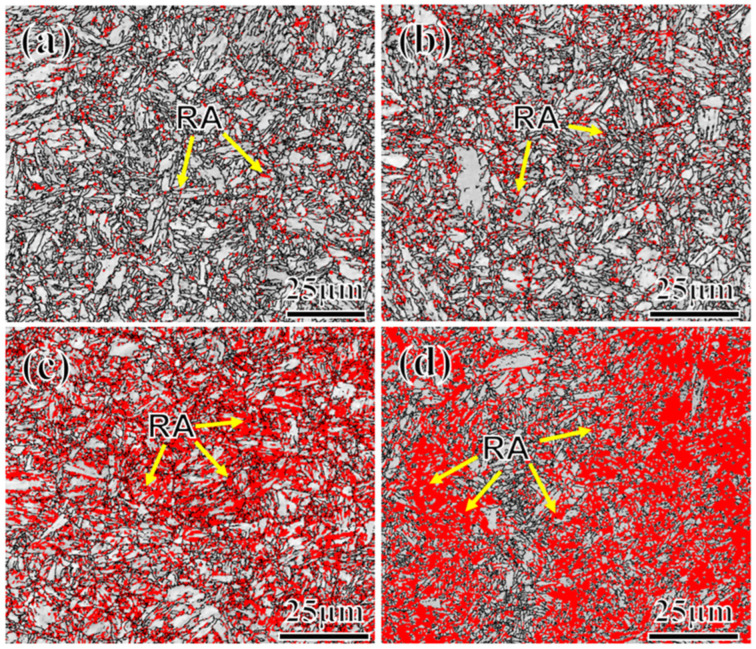
EBSD images for different Mn content of samples at 2Mn (**a**), 3Mn (**b**), 4Mn (**c**), and 5Mn (**d**)—all signaled by the yellow arrows.

**Figure 8:**
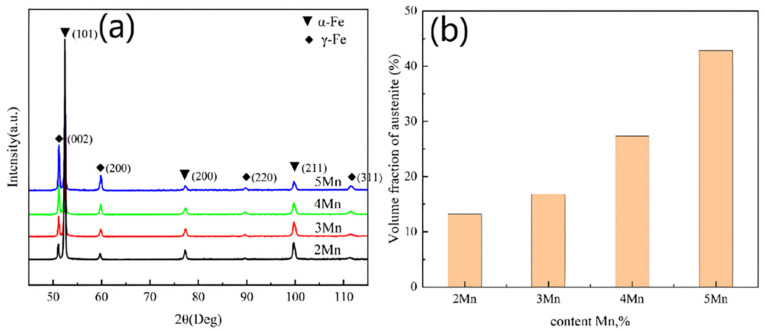
XRD patterns and austenite contents of steel with different Mn contents were tested: (**a**) XRD; (**b**) austenite content.

To further clarify the distribution and content of RA, EBSD analysis was conducted on the test steel, as shown in Figure 7. The red area in the figure indicates the presence of RA. The steel with higher Mn content exhibits a pronounced increase in the amount of RA. Regarding distribution, RA primarily occurs along the grain boundary and the lath boundary, which are the primary nucleation sites of RA, owing to the high energy present [35]. Regarding morphology, at a low Mn content (Figure 7a,b), RA appears mostly granular and strip-like, whereas, at higher Mn content (Figure 7c,d), blocky RA increases significantly in size. Considering stability from a volume fraction perspective, smaller-sized RA is more stable [36,37,38]. The statistics detailing volume fraction and grain size of the RA, measured via EBSD (test step 0.2 μm, pixel 600 × 600), are presented in Table 5, using four samples that use the same area (14,400 μm^2^). However, given the limited area sampled in EBSD, potential bias exists. Consequently, XRD is employed to further evaluate the austenite content. As shown in Figure 8a, the diffraction peaks of different Mn steels demonstrate an increase in the austenite diffraction peak with the increase in the Mn content; particularly, the integral intensity of the (002) diffraction peak increases significantly. Figure 8b displays the variation pattern of RA. The XRD test results generally align with the EBSD results, although slight deviations exist. This divergence is primarily attributed to the influence of EBSD resolution and sample surface quality. Owing to the large step size in EBSD, detecting smaller-sized austenite is challenging. Variations in the selected test area might also contribute to these deviations. In addition, it is also related to the selected test area. Even after tempering, variations persist within the microstructures of the material, which result in differences in the composition or distribution of phases across different regions of the material.

**Figure 9:**
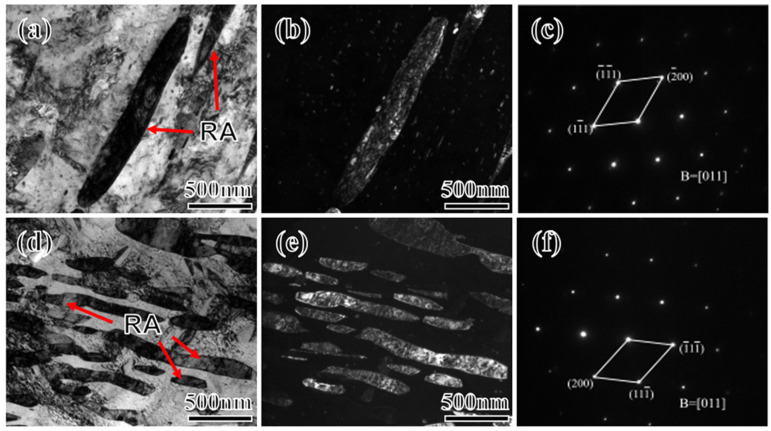
TEM images of different Mn content of samples at 2Mn (**a**–**c**) and 5Mn (**d**–**f**). [bright field (**a**,**d**); dark field (**b**,**e**); diffraction spots (**c**,**f**)] ‎(The red arrow points to RA).

**Figure 10:**
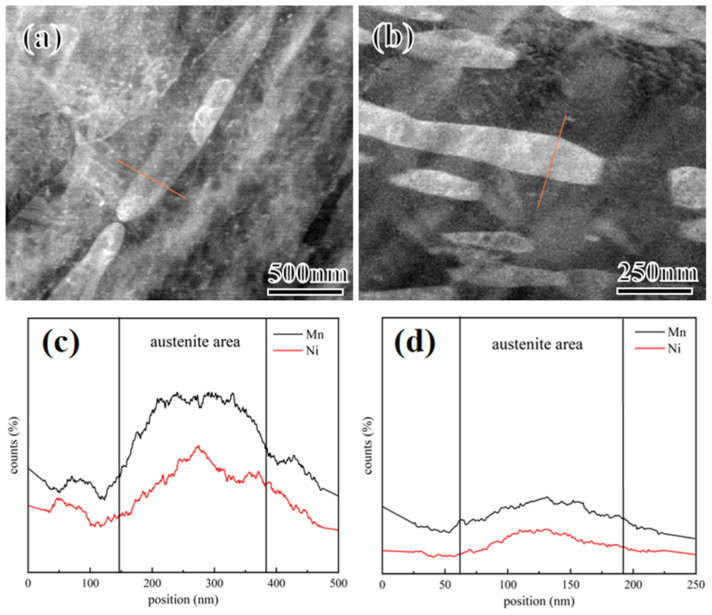
(**a**,**b**) STEM images of 2Mn (**a**), and 5Mn (**b**); (**c**,**d**) EDS showing the distribution of Mn, Ni elements in 2Mn (**a**) and 5Mn (**b**). Note: The red line in (**a**,**b**) indicates the position of the line sweep.

Figure 9 shows the transmission electron microscopy (TEM) images of 2Mn and 5Mn steels. As the Mn content increases, the amount of RA significantly increases, and the morphology gradually shifts from lath to a mix of lath and block [39,40]. The diffraction patterns of the RA in these samples predominantly indicate the γ-phase with an fcc structure. The distribution of RA exhibits a certain degree of orderliness, with most RA maintaining the same orientation as the original austenite [41]. Due to the higher tempering temperatures, the martensitic lath morphology is almost absent, and RA is uniformly distributed over the martensitic matrix. However, the formation sites of RA with different morphologies vary. In the 5Mn samples, a significant amount of blocky RA tends to form at the original austenite grain boundaries. The austenite nuclei at these grain boundaries maintain the Kurdjumov-Sachs (K-S) relationship with only one side of the matrix. These reverted austenite regions at the original austenite grain boundaries are enveloped by coherent and incoherent interfaces, leading to differences in surface energy and elastic strain energy on both sides, thereby facilitating the formation of blocky RA [42]. Analysis of the width of the lath RA in multiple TEM images reveals that the width increases from approximately 95 to 130 nm as the Mn content increases from 2 to 5Mn. The size of RA gradually coarsens, consistent with the EBSD statistical results. To examine the distribution of specific elements within the RA, STEM(scanning transmission electron microscopy)-EDS scanning was conducted on the RA in both 2 and 5Mn samples (Figure 10). According to Figure 10a,c, the Mn and Ni elements in 2Mn RA are significantly enriched, while the Mn and Ni enrichment of 5Mn is not high (Figure 10b,d). The high degree of segregation of Mn and Ni (which are stabilizing elements for austenite) indicates a higher stability of RA in 2Mn than in 5Mn.

## 4. Discussion

### 4.1. Effect of Mn Content on the Stability of RA and Work Hardening

With the increase in Mn content, the tensile strength and yield strength of the test steel increase, but the tensile strength increases more significantly. With the increase in the Mn content from 2 to 5%, the tensile strength increases by nearly 100 MPa. Concurrently, the yield ratio decreases from 0.96 to 0.90, indicating that the test steel with a higher Mn content exhibits better strain-hardening ability.

**Figure 11:**
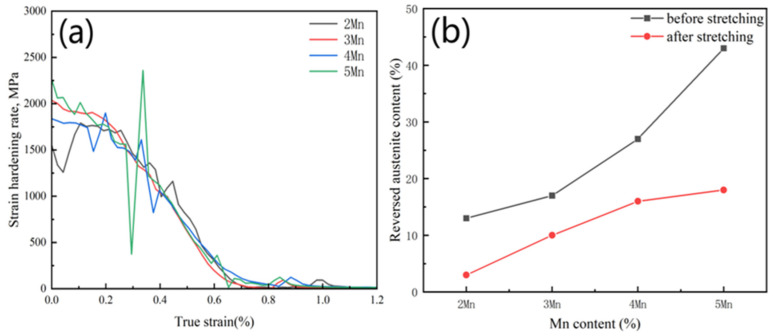
(**a**) Strain hardening rate curves of tested steels with different Mn content; (**b**) the change in austenite content before and after stretching.

The strain-hardening rate curve, depicted by the true stress–strain curve in Figure 11a, illustrates a gradual decrease in the work-hardening rate with the increasing true strain across all test steels. Notably, test steels with lower Mn content (2, 3Mn) exhibit a gentler strain-hardening rate curve, while those with higher Mn content (4, 5Mn) showcase significant changes in peak values. This discrepancy primarily arises from the presence of blocky RA with lower stability in the test steel with higher Mn content. With increasing strain, at critical stress levels, RA with similar stability exhibits the TRIP effect and transforms into FM under stress induction. This process releases a large amount of stress and causes a sharp decline in the strain-hardening rate curve. For example, in the case of 5Mn, a notably distinct peak alteration is observed around a true strain of approximately 0.3, indicating that the TRIP effect is significantly stronger. As strain progresses, additional RA with similar stability can transform FM, and a new round of the TRIP effect may occur. This process will occur repeatedly during the tensile deformation process, resulting in multi-peak strain-hardening behavior and sufficient TRIP effect [43,44,45]. The FM produced by phase transformation can increase the tensile strength of the test steel and improve its work-hardening ability [46].

According to the above results, to verify whether the TRIP effect occurs during the tensile process, samples from 2 to 5Mn post-tensile testing were selected to measure the austenite content using XRD. The results (Figure 11b) show a 10% and 24% decrease in austenite content in the samples 2Mn and 5Mn, respectively. This demonstrates the low stability of RA in 5Mn and the strong TRIP effect. Additionally, Table 4 indicates a significant increase in the size of RA in different Mn steels, from 2Mn (0.85 μm) to 5Mn (1.43 μm). The formation of FM following the phase transformation of the RA exerts static pressure on the untransformed RA, enhancing its stability. Under macroscopic stress, smaller-sized grain structures in the RA result in increased static pressure and higher stability [47].

According to the above analysis, with the increase in the Mn content (2–5%), the RA content increases significantly (12–41%), but the stability decreases continuously. Low-stability blocky RA can produce a stronger work-hardening ability. In addition, with an increase in the Mn content, although the TRIP effect becomes gradually significant, the plasticity is almost unchanged, and the percentage elongation is maintained at ~23.5%. This observation is mainly linked to the scope of the TRIP effect. Figure 11a shows that the strain range where the TRIP effect occurs in test steels (2–5Mn) with varying Mn content is nearly the same (i.e., before 1.2%). This indicates that the scope of the TRIP effect is relatively concentrated and does not span the entire plastic deformation process. Therefore, the effect on enhancing plasticity remains largely consistent.

### 4.2. Effect of Mn Content on Low-Temperature Toughness

The effect of Mn content on low-temperature toughness mainly manifests during the crack formation and propagation stages in low-temperature impact scenarios. The data in Figure 4e reveals variations in the crack formation and propagation work across test steels with different Mn contents. Notably, a substantial decrease in crack propagation work occurs under higher Mn content.

During the crack formation stage, 4Mn and 5Mn’s RA transforms into FM, due to the presence of numerous unstable RA particles in the impact process. While this phase change from face-centered cubic (fcc) to body-centered cubic (bcc) relieves stress and diminishes stress concentration, the rapid formation of a considerable volume of FM leads to significant volume expansion. This rapid formation also prompts collisions among FM, resulting in microcrack formation. This counteracts the toughening effect of phase transformation and even reduces toughness. In 2 and 3Mn, the RA content is lower and more stable than in 4 and 5Mn; moreover, a lower amount of FM is produced via phase transformation, and almost no microcracks are produced.

In the subsequent crack propagation stage, the stable lath-shaped RA in 2Mn and 3Mn can deflect cracks, which increases the crack propagation path and improves the crack propagation energy. However, in 4 and 5Mn, impeding crack propagation is difficult, owing to the small amount of stable RA. At a high Mn content, the Mn element is more likely to segregate and undergo embrittlement at the grain boundary, which also reduces the ability of the grain boundaries to impede cracks [48,49].

In addition to the influence of microstructure and element segregation on crack propagation, crystal defects also influence crack propagation speed. The fracture cleavage zone, observed via scanning electron microscopy (Figure 3), features a tongue-like pattern area (formed when the cleavage crack encounters a twin and causes secondary cleavage along the twin plane), which gradually increases with the increase in the Mn content. This trend indicates a growing number of deformation twins. Twinning, an intragranular defect, indicates the presence of a high-stress zone in the crystal, in which the hardness is high, and the crack is prone to rapid expansion along the twin boundary, thereby significantly reducing crack propagation energy [50,51,52]. Twinning is the deformation mode of crystals when intragranular slips are blocked. During impact, crystal deformation primarily occurs through slip and twinning. Slip generally prevails. The shear stress *T* that drives the movement of the slip system can be expressed by Equation (2) as follows:

*T* = *P* cos*θ*/*As* = *P*/*A*_0_ × cos*φ* cos*θ* (2)

where *φ* and *θ* represent the angle between the force axis and the normal direction of the slip surface and the angle between the force axis and the slip direction, respectively; *P/A*_0_ represents the yield strength; and cos*φ* cos*θ* denotes the orientation factor, which can be expressed by (3) as follows:

*Ms* = cos*φ* × cos*λ* (3)

Generally, *Ms* is referred to as the Schmidt factor. When both *φ* and *θ* are at 45°, the maximum product value is 0.5, and slip is most likely to occur at this orientation, known as the soft orientation. In other words, the higher the Schmidt factor, the easier the slip system starts. Therefore, the crystallographic data of the grains in each phase in the material can be detected via EBSD, and the Schmidt factor of the specific slip surface and slip direction of the crystal can be calculated to determine the ease of slip system initiation. Typically, the slip surface corresponds to the atomic close-packed surface, and the slip direction aligns with the crystal direction with the densest arrangement of atoms. The slip planes in bcc and fcc structures are {110} and {111}, respectively, while the slip directions are <111> and <110>. Table 6 presents the percentage of easy-to-operate slip systems (Ms > 0.45) on the slip surface and in the slip direction along the X, Y, and Z directions (loading directions of the sample platform). As the Mn content increases, the Schmidt factor in most directions exhibits a downward trend, particularly in the fcc crystal, in which *Ms* significantly decreases in the Y and Z directions. These findings indicate that, under higher Mn contents, it becomes more difficult for the crystal structure to deform through traditional slipping mechanisms. Instead, the material tends to undergo plastic deformation through twinning.

**Figure 12:**
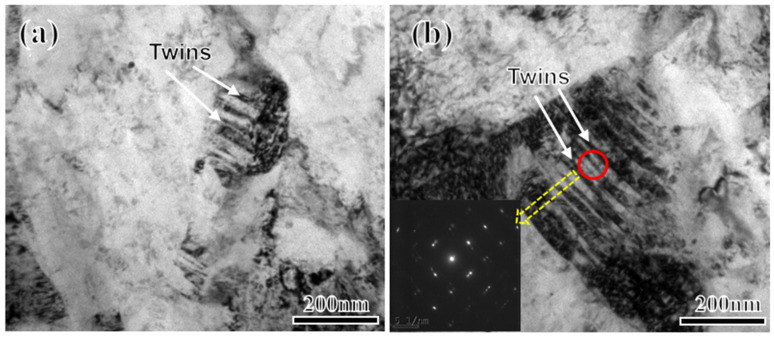
TEM images of 4Mn fracture deformation zone (**a**,**b**) after impact ‎(Red indicates the position of the diffraction spot, yellow arrow pointing to the diffraction spots).

From a different perspective, the grain size of RA significantly increases from 2Mn (0.85 μm) to 5Mn (1.43 μm). The finer grain size provides shorter slip paths, making plastic deformation more prone to occur through slipping. To verify the existence of deformation twins, a sample is extracted from the fracture deformation zone of 4Mn and observed via TEM. Figure 12 illustrates the twin structure present in 4Mn.

## 5. Conclusions

The purpose of this study was to explore the changing microstructure and properties of the test steel as the Mn content increased from 2 to 5Mn. Additionally, the analysis aimed to discuss the influence of Mn content. The main conclusions were as follows.

The increase in Mn content from 2Mn to 5Mn resulted in only a 33 MPa increase in yield strength, an approximately 100 MPa increase in tensile strength, and nearly unchanged elongation after fracture. Notably, 2Mn exhibited the best mechanical properties: a yield strength of 785 MPa, a tensile strength of 817 MPa, 23% elongation, and an impact work of 169 J at −84 °C.

With the increase in Mn content, the microstructure of the test steels gradually shifted from TM/F to RA and FM. Experimental findings indicated that the proportion of RA increased from 13 to 45% as the Mn content increased from 2 to 5Mn, accompanied by a significant decrease in stability.

As the Mn content increased, the formation and propagation energy of cracks decreased in the test steel. High Mn content, particularly in 4Mn and 5Mn samples, led to the production of substantial FM during impact. The resulting volume expansion caused microcrack generation owing to rapid collision, reducing the energy required for crack formation. Additionally, higher Mn samples generated more deformation twins during impact, significantly reducing the energy needed for crack propagation.

With the increase in the Mn content, the RA that can produce the TRIP effect in the tensile test gradually increased, leading to stronger work hardening. However, because phase transformation occurred mainly in the early stage of strain, the strain range for the occurrence of the TRIP effect was almost the same for steels with different Mn contents, resulting in almost the same plasticity enhancement effect.

## Figures and Tables

**Table 1:** Chemical composition of the experiment steels (mass fraction/%).

Steel	C	Si	Mn	Al	Ni	Cu	Nb	Ti
2Mn	0.046	0.25	2.09	0.042	3.53	2.1	0.018	0.020
3Mn	0.046	0.25	3.10	0.042	3.54	2.1	0.018	0.017
4Mn	0.043	0.25	4.11	0.038	3.60	2.11	0.018	0.018
5Mn	0.046	0.24	5.04	0.025	3.51	2.05	0.017	0.018

**Table 2:** The beginning temperature and the end temperature of austenite transition in the equilibrium state of the sample.

Phase Change Point/Sample	2Mn	3Mn	4Mn	5Mn
Ac3	790	770	770	758
Ac1	658	645	630	625
Ms	475	410	375	355
Mf	270	242	200	155

**Table 3:** The tensile properties of steel with different Mn contents were tested.

Sample	R_m_/MPa	R_p0.2_/MPa	A/%	Z/%	R_p0.2_/R_m_
2Mn	817 ± 3	785 ± 5	23.3 ± 0.3	79.0 ± 1.0	0.961
3Mn	840 ± 5	796 ± 8	23.5 ± 0.0	79.5 ± 0.5	0.948
4Mn	870 ± 2	794 ± 10	23.8 ± 0.3	78.5 ± 0.5	0.913
5Mn	910 ± 2	818 ± 4	23.3 ± 0.3	75.0 ± 0.5	0.899

**Table 4:** Volume fraction of reverse austenite (RA) and impact work of steel with different Mn content.

Sample	Volume Fraction of RA/%	Impact Work/J		
		−40 °C	−60 °C	−84 °C
2Mn	13	234 ± 30	191 ± 17	169 ± 5
3Mn	17	168 ± 10	154 ± 6	103 ± 20
4Mn	27	108 ± 7	65 ± 5	47 ± 3
5Mn	43	71 ± 4	53 ± 1	41 ± 4

**Table 5:** The content and size of reversed austenite (RA) were measured by EBSD in different Mn content test steels.

Sample	2Mn	3Mn	4Mn	5Mn
Volume fraction of RA/%	7	12	41	45
Grain size of RA/μm	0.85 ± 0.34	0.90 ± 0.39	1.38 ± 0.35	1.46 ± 0.50

**Table 6:** The proportion of easily activated slip systems in X, Y, and Z directions of crystals with different Mn contents (Ms > 0.45).

Sample	Crystal Structure	X	Y	Z
2Mn	bcc	55%	61%	60%
	fcc	62%	60%	63%
3Mn	bcc	58%	55%	62%
	fcc	62%	48%	46%
4Mn	bcc	57%	50%	61%
	fcc	51%	60%	58%
5Mn	bcc	53.8%	54%	64%
	fcc	60%	45%	36%

## Data Availability

All data included in this study are available upon request by contact with the corresponding author.
